# FIB-DIC Residual Stress Evaluation in Shot Peened VT6 Alloy Validated by X-ray Diffraction and Laser Speckle Interferometry

**DOI:** 10.3390/nano12071235

**Published:** 2022-04-06

**Authors:** Pavel A. Somov, Eugene S. Statnik, Yuliya Kan, Vladimir S. Pisarev, Svyatoslav I. Eleonsky, Dmitry Yu. Ozherelkov, Alexey I. Salimon

**Affiliations:** 1 HSM Laboratory, Skoltech, 121205 Moscow, Russia; pavel.somov@tescan.ru (P.A.S.); yuliya.kan@skoltech.ru (Y.K.); a.salimon@skoltech.ru (A.I.S.); 2 Central Aero-Hydrodynamics Institute Named after Prof. N.E. Zhukovsky (TsAGI), 140180 Zhukovsky, Russia; vsp5335@mail.ru (V.S.P.); juzzepka@mail.ru (S.I.E.); 3 Catalysis Laboratory, National University of Science and Technology “MISIS”, 119991 Moscow, Russia; d.ozherelkov@gmail.com

**Keywords:** residual stress, VT6 (Ti-6Al-4V) alloy, shot peening, X-ray diffraction $\sin^{2}\psi$, speckle interferometry, Ga-ion FIB-DIC

## Abstract

Ga-ion micro-ring-core FIB-DIC evaluation of residual stresses in shot peened VT6 (Ti-6Al-4V) alloy was carried out and cross-validated against other non-destructive and semi-destructive residual stresses evaluation techniques, namely, the conventional $\sin^{2}\psi$ X-ray diffraction and mechanical hole drilling. The Korsunsky FIB-DIC method of Ga-ion beam micro-ring-core milling within FIB-SEM with Digital Image Correlation (DIC) deformation analysis delivered spatial resolution down to a few micrometers, while the mechanical drilling of circular holes of ~2 mm diameter with laser speckle interferometry monitoring of strains gave a rough spatial resolution of a few millimeters. Good agreement was also found with the X-ray diffraction estimates of residual stress variation profiles as a function of depth. These results demonstrate that FIB-DIC provides rich information down to the micron scale, it also allows reliable estimation of macro-scale residual stresses.

## 1. Introduction

Shot peening is a cold processing technique that can significantly extend the working lifespan of components undergoing intense loads due to a favorable compressive layer of residual stresses (RS) and modifying the mechanical properties by hardening, removing corrosion or mechanical surface defects such as burrs or stripes after metalworking, etc.

Many various manufacturing processes used in aerospace, aviation, and automotive mostly create tensile stresses on the component surfaces. Typically, these methods include milling, drilling, turning, welding, heat treatment, and their combinations that provide tensile stress on the surface, thereby creating an ideal environment for crack initiation or stress corrosion formation, inevitably reducing the lifespan of the parts [1].

The key feature of the shot peening technique is protecting the material against mechanical corrosion and fatigue damage that frequently forms on the metal surface. Each shot plastically deforms the material during the processing and creates a compressive layer of the residual stresses on the surface that prevents crack initiation and propagation and significantly improves the material’s strength, reliability, and durability [2,3]. For instance, titanium blades that may be susceptible to different modes of damage under cyclic loading (high cycle fatigue, fretting fatigue) are often subjected to shot peening to extend their safe operating time. The control and monitoring of residual stresses in titanium blades is of great importance to assure the safety of aeroengines.

The steel or cast iron shots with a particle size of 0.5–2.0 mm are usually used in the shot peening. The optimal particle speed during the contact with the surface part is 50–70 m/s, the angle of shots incidence is 75–90°, processing time usually does not exceed 2–3 min, during which shots treat and penetrate surface layers at a depth of 0.2–0.4 mm. After that time, the ‘saturation’ effect is coming and the process does not cause noticeable changes in the quality and condition of the surface layers [4].

There are several methods that can measure and control the shot peening process, i.e., obtain profile and distributions of induced residual stresses. For instance, X-ray diffraction (XRD) is the most common non-destructive technique that can measure the absolute stress values and does not require a non-stress calibrated specimen. However, it has a main limitation connected to the significant penetration depth of the X-ray beam that affects the scale of the collected information. For example, conventional laboratory X-ray diffractometers typically probe samples to the depths up to ~20 µm that correspond to 1×…5× the average grain size for typical metals and alloys. Alternative methods such as hole drilling are destructive and also measure macroscopic residual stresses [5]. However, it is well-known that residual stress is an additive characteristic that separates the following three types of RS at the different scale levels, namely, $\epsilon =\epsilon_{I}+\epsilon_{II}+\epsilon_{III}$, where $\epsilon_{I}$ covers macroscopic RS, $\epsilon_{II}$ and $\epsilon_{III}$ are responsible for local microscopic (several grains) and intragranular RS, respectively [6,7].

In order to separate and extract RS from the microscopic scale level, the focused ion beam and digital image correlation (FIB-DIC) ring-core milling technique has been developed by Korsunsky et al. [8]. The Korsunsky method is based on stepwise material removal and obtaining relief strains by digital image correlation of scanning electron microscope (SEM) images.

Nowadays, the literature review returns only a single article of residual stress measurement on shot peened samples using FIB-DIC technique [9] where E. Salvati et al. compared obtained results with a simple numerical model of the eigenstrain cylinder. On the other hand, many new things have become known about the FIB-DIC method since then. For instance, (a) residual stresses depend on the scale level at which they are measured [9] and (b) there is correlation of determined residual stresses from deformation history [10]. Thus, a new aim arose to establish reliable experimental correlation of the FIB-DIC technique with other popular methods for conventional shot peening case. To perform the novel idea in this article, we measured residual stresses in VT6 (Ti-6Al-4V) alloy after shot peening using $\sin^{2}\psi$ X-ray diffraction, hole drilling with laser speckle interferometry monitoring, and Ga-ion FIB-DIC micro-ring-core milling methods at the different scale levels. Moreover, we characterized the microstructure and measured the hardness of the non-treated and shot peened surfaces by electron backscatter diffraction (EBSD) and nanoindentation techniques, respectively.

## 2. Materials and Methods

### 2.1. Sample Preparations

The 90 × 7.5 × 3 mm^3^ plates of VT6 alloy (produced by VSMPO-AVISMA Inc., Verkhnya Salda, Russia) were cut using an Electrical Discharge Machining (EDM) Mitsubishi MV-1200R (Abamet LLC, Moscow, Russia) from a drawn bar. The surfaces 90 × 7.5 mm^2^ were additionally mechanically polished to reach roughness $R_{a}=3.2\;µm$ and $R_{z}=25.2\;µm$. Polished samples were annealed at 630 °C for 2 h in vacuum to relax residual stresses introduced during polishing.

**Figure 1:**
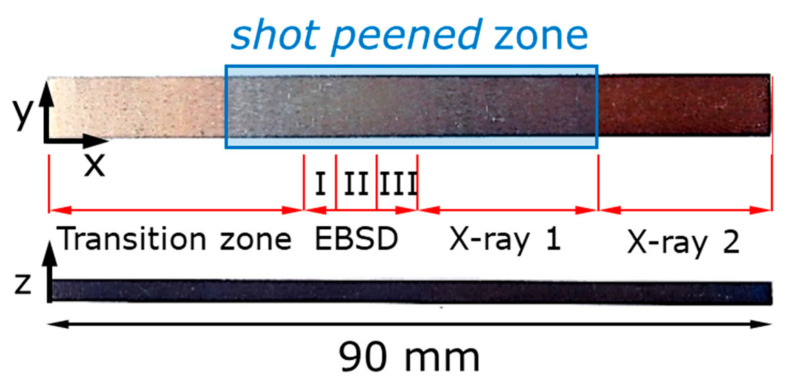
Initial specimen with the indicated coordinate system, dimensions, and regions of interest along which it was cut.

The steel balls (used in bearing) with diameter of 1.5 mm were accelerated with an ultrasonic agitating device to shot peen the surface of plates with colliding velocity of about 10 m/s. The construction of the shot peening chamber where plates were fixed at the roof provided the treatment in the middle zone approximately 50 mm long. The appearance of a titanium plate and the position of the shot peened window are given (blue frame) in Figure 1. One of shot peened plates was EDM sectioned into smaller parts (red lines) for various further analyses:

***Transition zone part*** included both shot peened and non-treated areas. The techniques of nanoindentation and FIB-DIC evaluation of RS were applied to probe the surface characteristics in terms of mechanical response and in-plane residual stresses.

***Three 7.5 × 5 × 3 mm^3^ parts*** (***I, II, III***) were carefully prepared for EBSD studies of microstructure and FIB-DIC evaluation of RS in deep layers under shot peened surface. The faces 7.5 × 3 mm^2^ (***Face I***), 5 × 3 mm^2^ (***Face II***) and 7.5 × 5 mm^2^ (***Face III***) were mechanically polished with a set of sandpapers, diamond pastes (1–3 µm), and colloidal silica, and then finished with ion gun Leica EM RES 102 (Leica Microsystems, Wetzlar, Germany) under the next regime: voltage 6 kV, current 2.6 mA, gun tilt 3°, and milling time 1 h.

***X-ray 1*** (shot peened surface) and ***X-ray 2*** (non-treated surface) parts were used to investigate RS in the surface layer with help of the X-ray ($\sin^{2}\psi$) method.

We discuss below the assumptions and limitations of the $\sin^{2}\psi$ method in respect of finding FIB-DIC.

### 2.2. X-ray Diffraction ($\sin^{2}\psi$) Method

**Figure 2:**
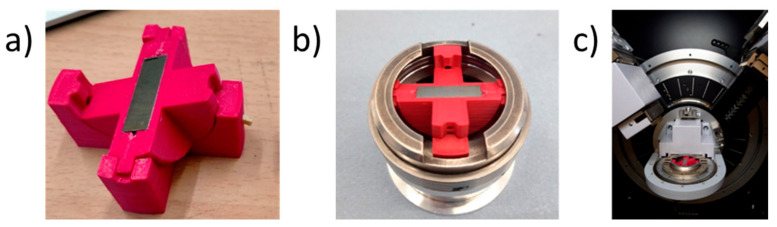
The details of X-ray diffraction ($sin^{2}\psi$) technique realization: (**a**) goniometer-like setup; (**b**) setup embedded into diffractometer grip; and (**c**) setup with grip into diffractometer.

A diffractometer XRD Bruker D8 Advance (Bruker AXS Inc., Madison, WI, USA) with Cu K_α_ wavelength of 0.154 nm dedicated for the study of powder samples was used to carry out $\sin^{2}\psi$ measurements. Due to the lack of options for side-tilting, a special sample holder (a single degree of freedom goniometer) was fabricated to allow sample inclination of 0, 10, 20, 30, and 40 degrees, whilst the X-ray source and detector were continuously scanned over the 2*θ* angle range 110–127 degrees. The tilting sample holder shown in Figure 2 was designed and 3D printed. By rotating the holder, it was possible to apply φ-tilting to determine residual stresses along the sample long axis, and ω-tilting for the transverse direction.

Acquired spectra were processed with the following data treatment pipeline: spectra fitting, background subtraction, and center peak positions for the calculation of $d_{HKL}$. In this case, residual stress can be determined from the slope of the d versus $sin^{2}X$ graph, where d is the measured lattice spacing and X is the tilt angle.

### 2.3. Mechanical Hole Drilling and Laser Speckle Interferometry

The hole-drilling method is a destructive technique for the experimental evaluation of residual stress in materials [11]. The removal of a certain amount of material with help of hole drilling (blind or through) causes the release of residual stress energy and local deformation—diameter increment or decrement. The detection of deformation response to drilling is a task requiring adequate sensitivity and accuracy since the strains to be measured are of about few thousandth and, therefore, few micrometer displacements must be detected for few millimeter big hole diameters. Drilling with a diameter 1.9 mm drill was applied. The distance between blind 2.5 mm-deep holes varied in the range of 10…15 mm.

The details of the experimental setup design applied for electronic speckle pattern interferometry (ESPI) were published previously [12,13]. Optical detecting system uses normal illumination (diode laser with wavelength of 532 nm) with respect to plane object surface and two symmetrical detectors (digital photo cameras) to record interferograms. Phase shift in respect to the surface interferogram before drilling forms specific patters—fringes the number of which is easily re-calculated to the diameter increment (with the respective sign) by means of multiplication by the factor λ/2sinθ, where λ is the incident illumination light wavelength, and θ is the angle between the incident and reflected beam.

### 2.4. Nanoindentation Technique

A NanoScan-4D system (FSBI TISNCM, Troitsk, Russia) was used to probe mechanical performance of VT6 sample at the surface using Oliver–Pharr method [14]. Diamond Berkovich indenter was used at the regime: linear increment of force to reach 3 N in 10 s, 0.6 s holding, and unloading with the same force rate. Young’s modulus and hardness were calculated with fused silica calibration and then processed to introduce work-of-indentation correction recommended for ductile materials [15].

### 2.5. FIB-SEM Studies: Microscopy, EBSD and FIB-DIC Ring-Core Drilling

A dual Ga-FIB FEG-SEM S9000 Solaris microscope (Tescan Orsay Holding, Brno, Czech Republic) was used for SE and BSE visualization of surface topography and EBSD (detector by Oxford Instruments NanoAnalysis and Asylum Research, High Wycombe, UK) characterization of structure—phase composition, grain size, shape, and orientation (texture) distribution with the help of the AZtec software (integrated software from Oxford Instruments supplied with the detector).

Areas for EBSD analysis were 200 and 400 µm^2^ with efficient pixel size of 1 µm^2^ for data collection at an acceleration voltage of 20 kV and beam current 10 nA.

The Korsunsky focused ion beam ring-core drilling method (FIB-DIC) is a destructive method for the evaluation of residual stresses at the micrometer scale [8]. Residual stresses of Type I, II, and III may be under some assumptions prescribed to the entities of different dimensional scale, namely, to an assembly of many grains, a single grain, and intragrain domains, respectively [7]. Averaged over a sufficiently large volume in a single grain, residual stresses of Type III return the estimation for Type II. In the same way, the average value of residual stresses of Type II taken from a sufficient number of single grains is equal to the residual stresses of Type I. These are the engineering scale residual stresses that directly correspond to the results of FE modeling based on continuous mechanics of solids. Thus, the representability and robustness of residual stress evaluation with the FIB-DIC ring-core method depend on the ratio between core diameter and grain size—when a number of or many grains are present inside a given core of a ring drill, the value of strain, appeared as a result of core unloading and respective residual strain release, corresponds to Type I residual stress in a certain locality. EBSD characterization of grain structure becomes a necessary preliminary step for correct selection of geometrical parameters of FIB ring-core drilling. The particular voltage and current of ion milling were empirically found and optimized in accordance with general recommendations given in [16] for this method to be the following: high voltage 30 kV, current 10 nA, tilt stage 55°, 10 µm inner diameter and 15 µm outer diameter of a ring, milling depth 1 µm/step, and dataset of 12 images.

### 2.6. DIC Processing

DIC (*iStress* software [17]) of image subsets is applied to detect the strains of a 10^−4^…10^−2^ for the core diameters of 1…10 µm. High resolution imaging at relevant magnification is required to reveal sufficient density of contrasting features inside the core for robust DIC. Sample preparation for EBSD analysis includes ion finishing that forms a specific surface topography enriched with noticeable grain boundaries and triple boundary joints—these surface features were customized for contrasting and purposeful selection of localities for FIB ring-core drilling.

## 3. Results and Discussion

### 3.1. Structure Characteristics

**Figure 3:**
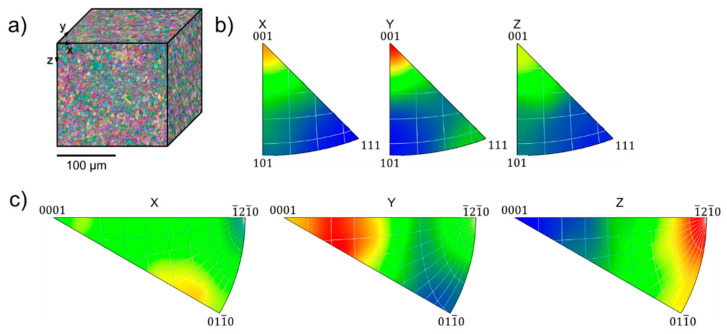
Structure characteristics of a shot peened VT6 samples: (**a**) pseudo-cubic of Euler color maps and IPF color maps for (**b**) β-Ti and (**c**) α-Ti, respectively.

Grain maps collected using EBSD scanning and contrasted with Euler colors for shot peened part are assembled in pseudo-cubic and represented in Figure 3a in the cross-sections corresponding to the axes *x*, *y,* and *z* designated in Figure 1. No difference in general appearance of grain patterns is detectable with the naked eye. Relatively small (5…10 µm) and equiaxial grain of α-Ti phase are found in all cross-sections, although pole charts clearly show strong texture with preferential orientation of (0001) planes in the samples. Grains of β-Ti phase (the volume fraction is less than 3%) are much smaller (0.5 …1 µm) and mainly located along grain boundaries and in few triple grain joints. No peculiarities were recognized in the subsurface layers of shot peened areas where the influence of severe plastic deformation caused by repetitive impacts might be anticipated.

### 3.2. Macroscopic Mechanical Hole Drilling

**Figure 4:**
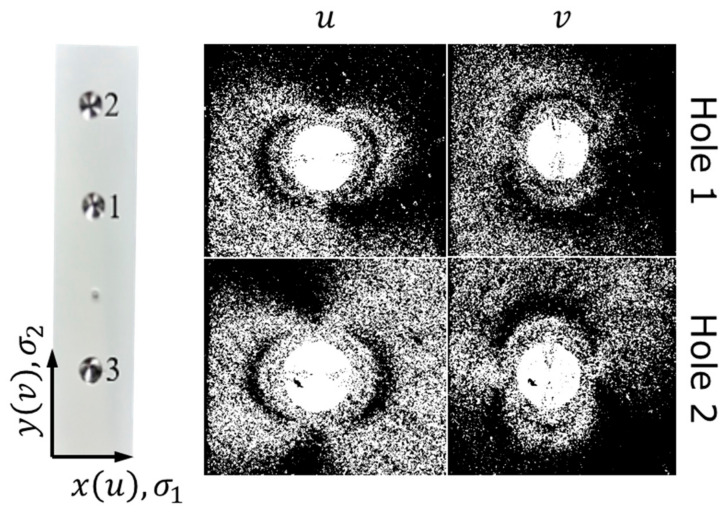
Appearance of drilled holes and interferograms for holes 1 and 2 with the corresponding coordinate system ($y\left(v\right)$ and $x\left(u\right)$) and stress notation ($\sigma_{1}$ and $\sigma_{2}$).

Three drilled holes are shown in Figure 4 and they characterize non-treated (1 and 2) and shop peened (3) areas.

**Figure 5:**
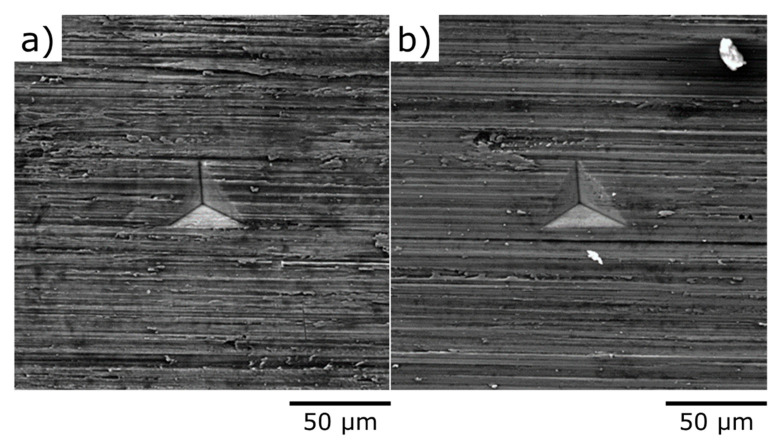
Appearance of VT6 sample surface (**a**) shot peened and (**b**) non-treated.

Relatively weak and almost isotropic residual tensile stresses were detected in the non-treated areas: 5 fringes along the u axis mean $\sigma_{1}$ = +83 MPa (Young’s modulus 115 GPa, Poisson’s coefficient 0.35) and 4.5 fringes along v correspond to $\sigma_{2}$ = +79 MPa. Surprisingly, no fringes could be imaged for hole 3 and other holes drilled in the shot peened area, what we relate to either instrumental issues (specific surface relief in the shot peened area, perhaps, makes fringes too noisy) or to the fact of averaging of tensile and compressive residual stresses when significant thickness comparable with the thickness of the plate is analyzed. This directly points to the necessity to apply techniques relevant to smaller thickness in terms of spatial resolution especially when relatively thin layers are affected. From the surface appearance images taken in the ***Transition zone*** in the vicinity of about 5 µm-deep indents (Figure 5), one can conclude that the system of scratches remained after mechanical polishing is slightly modified towards less regular relief with the signs of deformation and smashing of asperities. The tracks of scratches are visible down to the bottom of indents (about 5 µm). Nevertheless, scratches are clearly recognizable suggesting that severe plastic deformation did not propagate much deeper than 5 µm, while the general thickness of the shot peening affected zone is believed to be bigger by a factor of 3…5. The resolution of residual stresses in depth, therefore, might have to be comparable with this value, i.e., to be about 20 µm, what fairly corresponds to the estimation based on the average grain size—few grain diameters also return the scale parameter of about 20 µm.

### 3.3. Hardness Measurements

**Figure 6:**
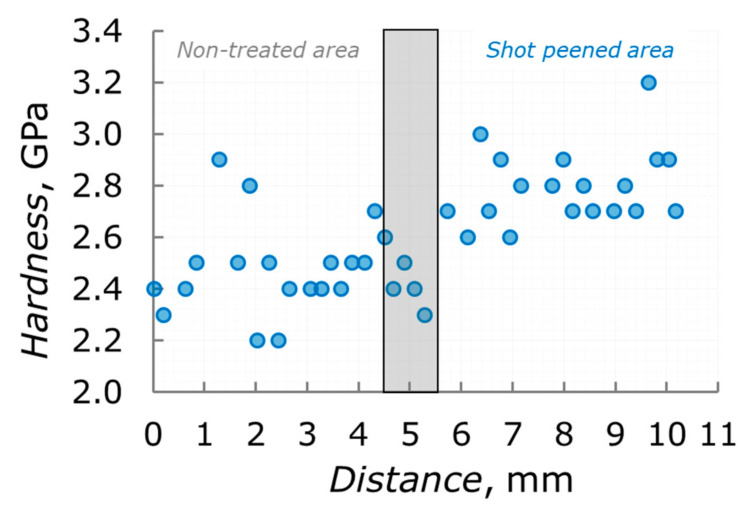
Hardness (blue circles) of VT6 alloy in the ***Transition zone*** sample.

Probing of mechanical response at the surface shows some hardening of shot peened materials against a non-treated one, namely, 2.79 ± 0.14 GPa against 2.48 ± 0.17 GPa as seen from Figure 6.

Some work hardening due to the severe plastic deformation is supposed to be the main reason for this difference in hardness. As we discussed above, it does not seem that layer of plastic deformation caused by shot peening may propagate to the depth for more than the first tens of micrometers.

### 3.4. X-ray Diffraction Evaluation of Residual Stresses

**Figure 7:**
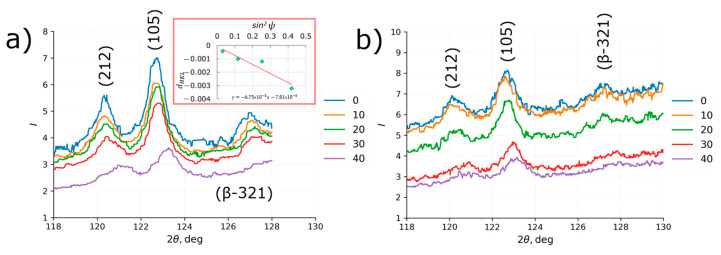
X-ray diffraction spectra collected for ψ = 0, 10, 20, 30, and 40 degrees from ***X-ray 1*** specimen aligned along the sample’s (**a**) x axis and (**b**) y axis. Inset is a plot of $d_{HKL}$ vs. $\sin^{2}\psi$ linearization plot.

Typical X-ray spectra acquired for ψ = 0, 10, 20, 30, and 40 degrees as well as d against $\sin^{2}\psi$ linearization plot are demonstrated in Figure 7. It is worth noting that the gradual shift of peak with the increase in ψ is easily noticeable with the naked eye suggesting good sensitivity of the method even when noisy spectra are analyzed such as those in Figure 7a. Strong dependence of the signal-to-noise ratio on the sample orientation is obviously related with the texture that was discovered by EBSD.

The data on peak shift and estimates of residual stresses for two $\left(hkl\right)$ crystallographic directions in α-Ti and one $\left(hkl\right)$ in β-Ti phases are accumulated in Table 1. Compressive residual stresses along both *x* and *y* sample’s axis as strong as −1000 MPa have been detected at both sides of the sample where the shot peening treatment was applied—shot peening gives good uniformity and reproducibility of treatment. Compressive residual stresses along (212) direction seem to be at least 30% stronger than those along the (105) (close to (001)) direction also suggesting predominant concentration of eigenstrains within non-basal planes of hexagonal lattice of α-Ti. No reliable conclusions can be made on residual stresses due to the wide spread (values differing by more than a factor of 3) of measured values along different sample axes and at different sample sides.

The data on residual stresses in the non-treated area are not (or much less) statistically reliable; however, relatively small tensile stresses appear to be present. These might be inherited from previous sample treatments which affected the material, including forging/rolling, annealing, cutting, and mechanical polishing. The presence of small tensile residual stresses is detected by the mechanical hole drilling method, suggesting their preservation both at the surface and in the deep subsurface layers.

Residual stresses in the shot peened area show weak dependence on the crystallographic orientation $\left(hkl\right)$ of grains assuming that residual stress is relatively isotropic in the surface layer regardless of the strong texture detected by EDSD. It is likely that severe plastic deformation activates many slip systems in α-Ti crystalline lattice including non-basal planes—that averages the stress state formed in the surface layer.

The $\sin^{2}\psi$ method owes its popularity to the advantage of its convenient implementation within laboratory diffractometers. However, it is also subject to a number of significant shortcomings that have been critically discussed previously [18].

Four assumptions underlying conventional the $\sin^{2}\psi$ method are as follows:

A1. The penetration of the X-ray beam is so shallow that the material interrogated can be thought to be in the state of plane stress.

A2. The grain ensemble contributing to the diffraction peak is representative of the state of deformation in the sample as a whole.

A3. Despite the polycrystalline nature of the sample, it can be represented correctly by a homogeneous elastic continuum.

A4. Even in the presence of plastic deformation which causes residual stresses, correct values of the diffraction elastic constants (DEC’s) can be identified, and Hooke’s law can be applied to relate stresses and strains.

Whilst it is important to emphasize that all these assumptions are approximate and do not hold in practice, for the present purposes it is worth focusing attention on Postulate A3 that deals with data interpretation based on continuum homogeneous elasticity. In fact, polycrystalline samples consist of multiple grains of different orientations with different elastic and plastic properties. The strains in this grain ensemble are different from a uniform continuum. Furthermore, sample tilting leads to data collection from different grain groups, which may lead to significant deviations that affect the results.

The conclusion is drawn at this stage that the outcomes of the $\sin^{2}\psi$ method are likely to be subject to significant aberrations that may reach 20–30% systematic or random error, and need to be supported with measurements using other independent methods to improve reliability of interpretation.

### 3.5. Residual Stresses atthe Microscopic Level—FIB-DIC Evaluation Results

**Figure 8:**
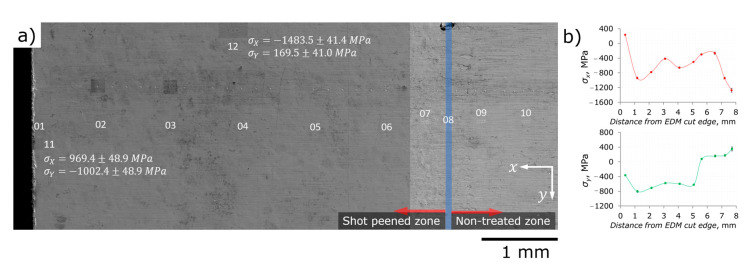
FIB-DIC evaluation of RS at the surface in the ***Transition zone*** specimen: (**a**) location of FIB-DIC probing; (**b**) profile of RS along the x and y axes.

*Shot peened surface.*Figure 8a is a superposition of the ***Transition zone*** specimen SEM image and designations of locations where FIB core-ring drilling was used to probe residual stresses at the surface of shot peened and non-treated areas. Locations $\left(01\bar{1}0\right)$ are generally aligned along the x axis and in the middle of the sample. Locations 11 and 12 are as close as 100 µm to the edges—11 is close to EDM cut, while 12 corresponds to the longest edge of sample in the shot peened area. Figure 8b depicts the estimates of residual stresses which were present at the sample surface before drilling. The series of indents (hardness measurements) is also visible in Figure 8a.

A number of observations can be carried out from a fast glance:

- Locations 02…06 show approximately equal values of compressive residual stresses $\sigma_{x}$ and $\sigma_{y}$ in both principal directions in the range of −800…−400 MPa. This is in good agreement with the estimations of the X-ray measurements.
- Locations 07…10 as well as 11 and 12 where some peculiarities of sample geometry or treatment are undoubtedly present—edges or boundary of shot peened and non-treated areas—reveal strong asymmetry of residual stresses. This asymmetry means both different signs of stresses and enormous absolute difference in the values of stresses—for at least 1600 MPa. Location 01 also shows different signs of $\sigma_{x}$ and $\sigma_{y}$, but absolute difference is somewhat smaller—of about 600 MPa.
- At the edges, tensile stresses exist.
- The boundary between shot peened and non-treated areas from a residual stresses’ point of view occurs much broader than that which can be recognized from the difference in surface appearance. Considering the locations 07…10 as belonging to the boundary area, the width of this area may be assessed as 2 mm (2 shot peening balls diameters).

Emergence of tensile residual stresses at these peculiarities is stated of great importance—the promotion of nucleation and growth of fatigue cracks becomes possible in some directions even for shot peened parts, which are theoretically protected against cracking phenomena due to the introduction of compressive stresses. EDM cutting is considered as the least invasive macroscopic technique [19], i.e., it introduces minimal damage to the least depth material. Our data prove that EDM cutting is not recommended *after* shot peening treatment—we believe that the redistribution/relaxation of residual stresses may cause very detrimental effects.

**Figure 9:**
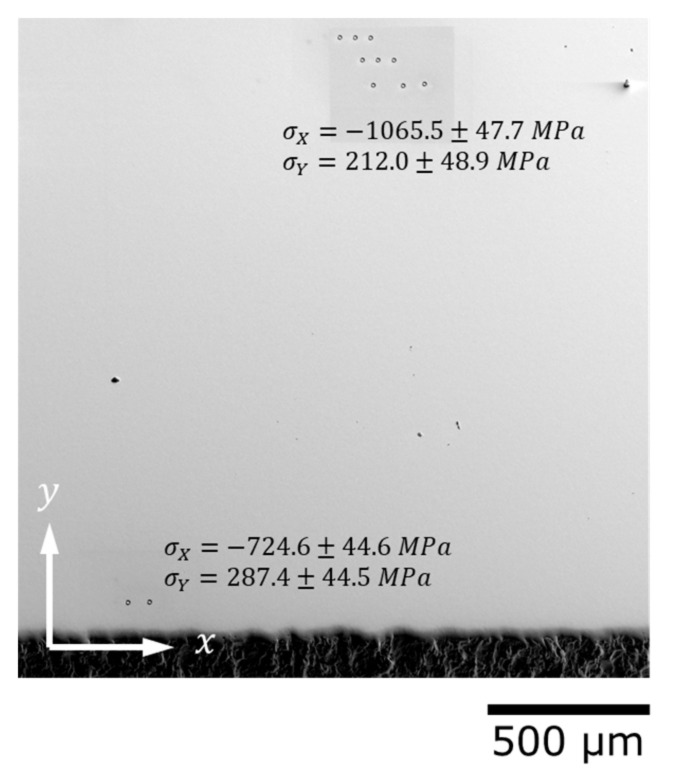
RS under shot peened area—***Face III*** 7.5 × 5 mm^2^.

***Face III* part, shown in Figure 9,** is parallel to the shot peened surface and lays at the depth of about 80 µm. As one can see, the accurate preparation of the sample for EBSD studies fully eliminates surface scratches as well as all signs of shot peening. The sequence of polishing operations does redistribute residual stresses present before polishing and introduces new residual stresses; on the other hand, we believe that the polishing techniques applied [20] are much less intensive than shot peening and no substitutional changes are caused by polishing as it is. The redistribution and relaxation of residual stresses due to the elimination of stressed surface layers seem to be predominantly responsible for the appearance of tensile in-plane residual stresses. The contribution of out-of-plane (which are discussed below) is suspected to introduce harmful tensile stresses, which may facilitate surface crack nucleation and growth. Therefore, polishing is also not recommended *after* shot peening treatment.

**Figure 10:**
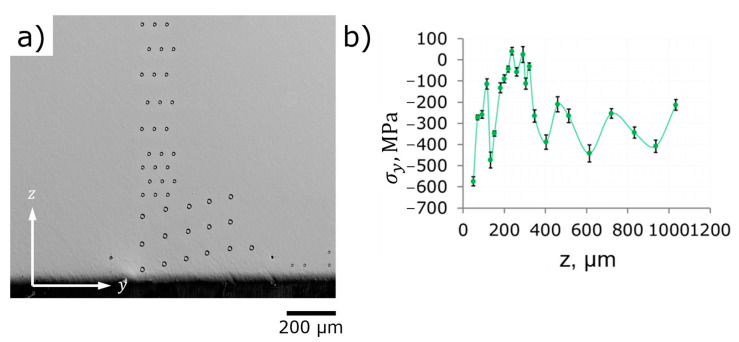
Depth distribution of RS in shot peened area (the middle of sample): (**a**) SEM image of probing FIB core-ring holes at ***Face I*** 7.5 × 3 mm^2^; (**b**) profiles of $\sigma_{y}$ and $\sigma_{z}$ against depth.

*Profiling of residual stresses in depth*. Figure 10a shows the locations of probing FIB core-ring holes—different ring diameters, distances, and number of repetitions were applied in order to reach satisfactory spatial resolution and statistical fidelity within subsurface layers and to optimize ion drilling time and Ga-consumptions. Subsurface $\sigma_{y}$ stress profile (in-plane stresses, parallel to shot peened surface) starts from the depth of 40 µm with the value of −600 MPa. The estimation for the same orientation of residual stresses at the surface is −500 MPa and it perfectly agrees with the expected decrease towards deeper layers.

In contrast to the anticipated RS profile, the maximum that is reached at the distance of about 300 µm from the surface gives near-zero values, while the steady level of RS that starts at the distance of 400 µm from the surface corresponds to the compression zone with stable residual stresses in the range −400… 200 MPa.

**Figure 11:**
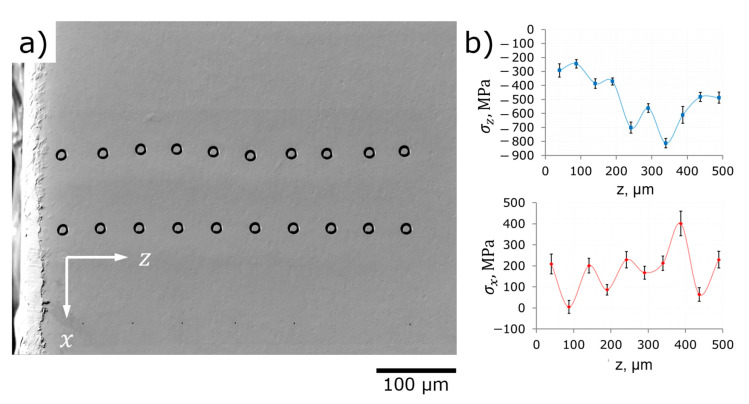
Depth distribution of RS in shot peened area (the edge of sample): (**a**) SEM image of probing FIB core-ring holes at ***Face II*** 5 × 3 mm^2^; (**b**) profiles of $\sigma_{x}$ and $\sigma_{z}$ against depth.

Subsurface $\sigma_{z}$ stress profile (out-of-plane stresses, perpendicular to shot peened surface) is asymmetrical to the $\sigma_{y}$ stress profile—modest tensile stresses at shallow depth, minimum stresses at the depth of about 300 µm from the surface (about −400 MPa), and stabilization at near-zero stress values at the depth of about 400 µm from the surface. Out-of-plane stresses cannot be measured using X-ray diffraction ($\sin^{2}\psi$) or hole drilling methods and they are commonly considered as null at the surface from a static mechanics point of view. FIB core-ring method, nevertheless, detects significant (+300 MPa) tensile stresses in the subsurface layer at the depth of 40 µm. This fact should not be neglected since the same phenomenon is reproducible at ***Face II*** as demonstrated in Figure 11.

Subsurface $\sigma_{x}$ stress profile (in-plane stresses, parallel to shot peened surface) build at the sample’s edge is dramatically different from that in the middle of the shot peened area suggesting specific conditions of shot peening treatment at the edge. No maximum of residual stresses at the depth of about 300 µm was found, in contrast, a minimum (−800 MPa) was discovered. In general, ***Face II*** shows relatively strong (−800…−200 MPa) compressive residual stresses at the distances 40…500 µm from the surface. This also corresponds to the estimations for surface in-plane residual stresses obtained at the sample’s edge (location 12 in Figure 8a).

Out-of-plane residual stresses $\sigma_{z}$ are nill at the shallowest depth and then raise with the distance from the surface to the values in the range of 100…400 MPa with no distinct tendency. The origin of these stresses is not fully clear and they can be thought as the relicts of previous treatments, e.g., plate rolling.

## 4. Conclusions

The Korsunsky FIB core-ring drilling technique of residual stress evaluation has been an established approach for about 15 years. This method has enabled the correlation and comparison of measurements conducted at dramatically different dimensional scales, from micrometers to millimeters. However, methodological issues remain concerning the representativity of microscopic volumes for measurements of Type I (macro-scale) residual stresses and averaging of residual stresses over grain assemblies.

For a comparative case study, in this paper we selected a well-researched example of shot peening treatment for the introduction of compressive residual stresses into surface layers of parts susceptible to fatigue. To evaluate the residual stresses induced in shot peened VT6 (Ti-6Al-4V) specimens, macroscopic destructive (hole drilling) and non-destructive (X-ray diffraction) methods were used for the validation of FIB-DIC ring-core drilling results.

The following conclusions are drawn:

(1) It is hard to adequately study thin and slim shot peened samples by a macroscopic hole drilling method due to big size of drills because thin subsurface layers seem to be held out of relaxation by core layers of the material.
(2) The X-ray diffraction method demonstrated a good agreement with the FIB core-ring drilling approach. Both methods returned high values of compressive residual stresses of about −500 MPa.
(3) It was found that the FIB core-ring method is a favorable method of residual stress evaluation when fine details (as well as thin and slim samples or small cross-sections) of residual stress spatial distribution or depth profiles are wanted to be characterized. It also allows the detection of relaxation and stress redistribution effects close to the geometrical or physical peculiarities of a sample—edges, cuts, and transition zones.
(4) We proved that the FIB core-ring method fairly agrees with the X-ray diffraction method (own data) and hole drilling method (literature data) in terms of signs, values, and depth profiles.

Finally, we observed interesting features in the depth variation of the out-of-plane residual stresses. Tensile stresses found in shallow subsurface layers may promote delamination cracking and surface degradation. This is an interesting topic for future research in this field.

## Figures and Tables

**Table 1:** Residual stresses in ***X-ray 1*** (shot peening) and ***X-ray 2*** (non-treated) specimens of VT6. The estimations of the residual stress for correlation coefficient below 0.85 (absolute value) are given in Italic typeset.

Sample	$\left(hkl\right)$	Peak Position, 2θ Degree					Residual Stress, MPa	Correlation Coefficient
		ψ=0°	ψ=10°	ψ=20°	ψ=30°	ψ=40°		
**X-ray 1** (along the sample’s x axis)	(212)	120.2	120.2	120.3	120.7	120.8	−1330.1	0.97
	(105)	122.6	122.6	122.7	122.9	123.1	−964.6	0.98
	(β-321)	127.1	127.1	127.3	127.4	127.6	−907.8	0.99
**X-ray 1** *back side* (along the sample’s x axis)	(212)	120.3	120.3	120.3	120.6	120.9	−1318.9	0.97
	(105)	122.6	122.6	122.6	122.8	123.1	−1011.3	0.95
	(β-321)	127.3	127.0	127.2	127.2	127.6	−522.6	0.73
**X-ray 1** (along the sample’s y axis)	(212)	120.4	120.3	120.5	120.4	121.1	−1273.1	0.86
	(105)	122.7	122.7	122.8	122.8	123.2	−931.9	0.89
	(β-321)	126.7	127.1	127.2	127.2	127.8	−1794.1	0.91
**X-ray 1** *back side* (along the sample’s y axis)	(212)	120.3	120.3	120.3	120.6	120.1	−1478.4	0.98
	(105)	122.6	122.6	122.7	122.9	123.2	−1002.4	0.96
	(β-321)	127.1	127.1	127.1	127.5	127.7	−1042.8	0.97
**X-ray 2** (along the sample’s x axis)	(212)	121.0	120.9	120.8	120.9	120.9	118.9	0.34
	(105)	123.1	123.1	123.1	123.1	123.1	−34.3	0.40
	(β-321)	127.7	127.6	127.7	127.6	127.7	32.3	0.06
**X-ray 2** (along the sample’s y axis)	(212)	120.7	120.7	120.7	120.7	120.8	237.3	0.86
	(105)	122.9	122.9	122.9	122.9	122.9	291.0	0.87
	(β-321)	127.5	127.6	127.5	127.5	127.6	97.3	0.21

## Data Availability

Data presented in this article are available on request from the corresponding author.
